# Association between neutrophil percentage-to-albumin ratio (NPAR) and sarcopenia in individuals with arthritis: a cross-sectional study

**DOI:** 10.3389/fmed.2025.1602224

**Published:** 2025-12-09

**Authors:** Yancun Li, Ping Jiang

**Affiliations:** 1 College of Traditional Chinese Medicine, Shandong University of Traditional Chinese Medicine, Jinan, Shandong, China; 2 First Clinical Medical College, Shandong University of Traditional Chinese Medicine, Jinan, Shandong, China

**Keywords:** arthritis, sarcopenia, neutrophil percentage-to-albumin ratio, National Health and Nutrition Examination Survey, cross-sectional study

## Abstract

**Objective:**

Sarcopenia is a highly prevalent comorbidity in individuals with arthritis and a significant contributor to disability. Persistent chronic inflammation is a crucial common feature of both sarcopenia and arthritis. The neutrophil percentage-albumin ratio (NPAR) is a readily available and affordable marker for observing inflammation and nutritional status, used in the assessment of various diseases. The objective of this study was to explore the association between NPAR and sarcopenia in patients with arthritis.

**Methods:**

This study utilized data from the National Health and Nutrition Examination Survey (NHANES) 1999–2006 and 2011–2018, selecting adult participants with arthritis (including osteoarthritis, rheumatoid arthritis, and other types) and retaining only those with appendicular lean mass measured by dual-energy X-ray absorptiometry (DXA) and complete NPAR data. A multivariate logistic regression analysis was conducted to investigate the relationship between NPAR and arthritis with sarcopenia, followed by restricted cubic spline analysis (RCS). Subgroup analyses further explored the associations between NPAR and other covariates. Receiver operating characteristic (ROC) curves were utilized to compare the diagnostic value of NPAR, as a composite indicator, with that of individual indicators for arthritis with sarcopenia.

**Results:**

After adjusting for all potential confounders, a positive correlation was observed between NPAR and the prevalence of arthritis with sarcopenia. For each one-unit increase in NPAR, it was associated with a 22% increase higher odds of sarcopenia prevalence [odds ratio (OR): 1.22, 95% confidence interval (CI): 1.09, 1.36]. Among different types of arthritis, NPAR demonstrated a significant positive correlation with the prevalence of sarcopenia in both rheumatoid arthritis (RA) (OR: 1.36, 95% CI: 1.07, 1.74) and other types of arthritis (OR: 1.37, 95% CI: 1.16, 1.63), whereas this correlation was not evident in osteoarthritis (OA). Additionally, in ROC analysis, the Area Under the Curve (AUC) for NPAR as a composite indicator (0.587) was higher than that for neutrophil percentage (AUC = 0.571) and albumin (AUC = 0.549) as individual indicators.

**Conclusion:**

A higher NPAR value is associated with a higher prevalence of sarcopenia among individuals with arthritis. Furthermore, this composite indicator exhibits better diagnostic value compared to individual indicators.

## Introduction

The prevalence of arthritis, a common chronic inflammatory disease, is on the rise every year, with one prediction suggesting that by 2040, nearly 49 percent of the US population will have arthritis (1). Osteoarthritis (OA) and rheumatoid arthritis (RA), as common types of arthritis, differ in their pathogenesis. OA is mainly characterized by progressive degeneration of articular cartilage, formation of secondary osteophytes and subchondral remodeling (2). In contrast, RA is a systemic autoimmune disease with persistent chronic inflammation (3). However, the pathogenesis of both is closely related to the inflammatory response. Decreased muscle mass is a common complication of arthritis, including common OA (4), RA (5), and psoriatic arthritis (6). Inflammation also plays an important role in the development of sarcopenia (7). Chronic inflammation mediated by cytokines such as tumor necrosis factor (TNF) and interleukin-6 (IL-6) can lead to abnormal muscle homeostasis, for example, by exacerbating muscle protein breakdown (8). Sarcopenia, which involves loss of muscle mass and strength, is a progressive, systemic skeletal muscle disease that is strongly associated with adverse outcomes such as falls, weakness, and death (9), and severely affects patients’ quality of life. Therefore, it makes sense to identify and assess muscle mass and the risk of sarcopenia in patients with arthritis and intervene to manage it.

Dual-energy X-ray absorptiometry (DXA) represents a pivotal diagnostic tool in the assessment of sarcopenia (10). This technique offers exceptional accuracy in quantifying whole-body lean body mass. The ratio of appendicular skeletal muscle mass (ASM) to body mass index (BMI) serves as a valuable indicator in determining the presence of sarcopenia (11). Conversely, in individuals with arthritis, who frequently experience pain, the utilization of grip strength measurements may introduce inaccuracies due to the discomfort associated with the test (12). Consequently, there remains a pressing need for the identification of novel and readily accessible predictors of sarcopenia.

The neutrophil-to-albumin ratio (NPAR) is an easily obtainable and effective composite indicator that reflects both inflammatory status and nutritional levels within the body (13). Neutrophils play a crucial role in orchestrating inflammatory responses and immune homeostasis; they have been implicated in promoting muscle atrophy (8), and deregulated neutrophil activation is also considered closely associated with various autoimmune diseases, such as RA (14). Serum albumin is vital for maintaining nutrition and osmotic pressure in the human body, and studies have found decreased albumin levels in patients with RA and OA (15). Research has further demonstrated that NPAR exhibits risk correlation and effective predictive value in conditions such as diabetes (16), cardiovascular diseases (17), metabolic syndrome (18) and infectious diseases (19). Unlike single biomarkers, NPAR integrates inflammatory and nutritional parameters, aligning with the multifactorial pathogenesis of sarcopenia in arthritis. Critically, this relationship remains underexplored in current literature.

Since sarcopenia in arthritis is influenced by both inflammatory and nutritional factors, we hypothesize that the NPAR is related to the prevalence of sarcopenia among individuals with arthritis. In this study, we employed data from the National Health and Nutrition Examination Survey (NHANES) to investigate the association between them.

## Materials and methods

### Study population

**FIGURE 1:**
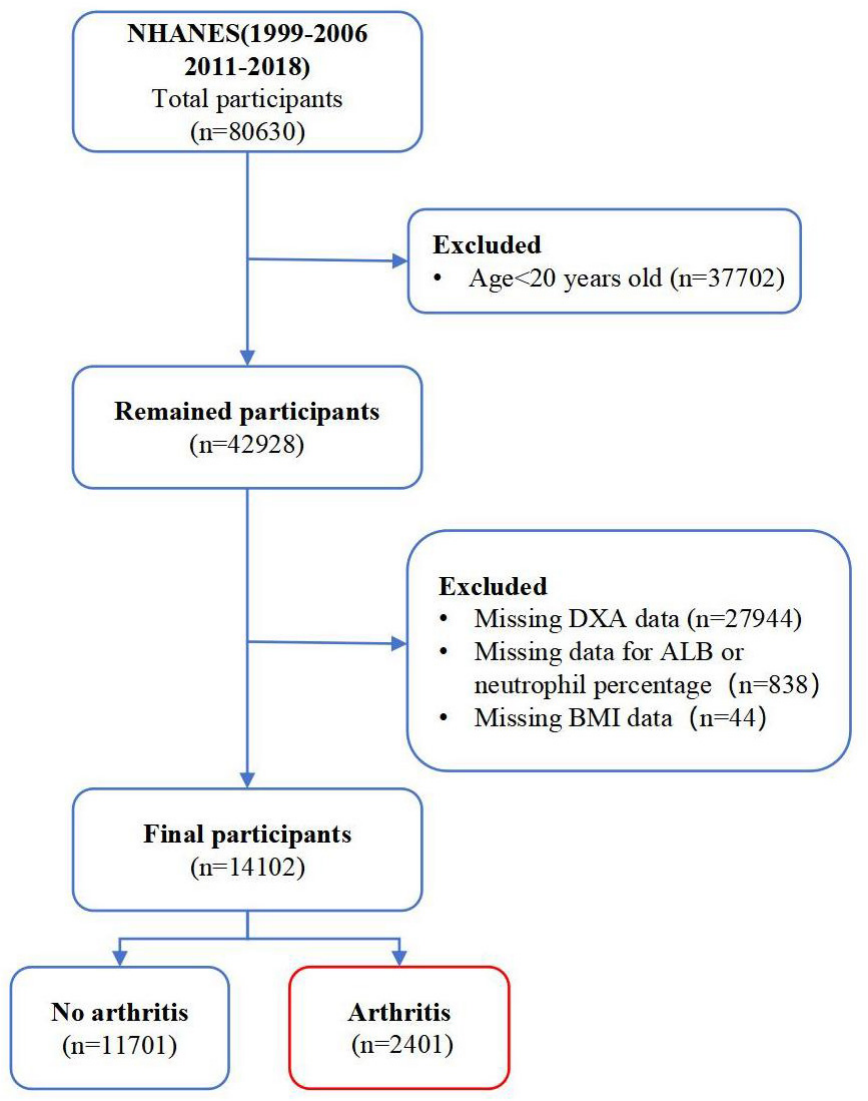
Flowchart of participant inclusion criteria. DXA, dual-energy X-ray absorptiometry; ALB, albumin; BMI, body mass index.

This study is a cross-sectional research utilizing data sourced from the NHANES, conducted by the National Center for Health Statistics (NCHS). The NHANES is primarily designed for assessing the health and nutritional status of the general population in the United States. This study follows the cross-sectional research guidelines outlined by the Strengthening the Reporting of Observational Studies in Epidemiology (STROBE) (20). All participants provided informed consent. The study is based on data from NHANES 1999 to 2018, excluding the cycles from 2007 to 2010 due to the lack of DXA data, resulting in the inclusion of a total of eight cycles. Initially, a total of 80,630 individuals were recruited. Among them, 37,702 participants under 20 years old were excluded, 27,944 participants with incomplete appendicular skeletal muscle mass data from DXA (pregnant women were not included in the DXA testing), 838 participants lacking data on albumin or neutrophil percentage, and 44 participants with incomplete body mass index (BMI) data were also excluded. Ultimately, a total of 14,102 participants were included, consisting of 11,701 individuals without arthritis and 2,401 individuals with arthritis (Figure 1).

### Calculation of NPAR

NPAR is the ratio of the percentage of neutrophils (as a proportion of the total white blood cell count) to serum albumin in human blood, with all data originating from laboratory tests conducted by NHANES. It is calculated using the following formula: NPAR = [Neutrophil Percentage (%) × 100]/Albumin (g/dL) (21).

### Definition and classification of arthritis

The inclusion of patients with arthritis is primarily determined by their responses to the “Medical conditions” questionnaire in NHANES, specifically the question: “Has a doctor or other health professional ever told you that you have arthritis?,” with “Yes” responses qualifying for inclusion. Classification is based on participants’ answers to the question: “Which type of arthritis was it?,” with responses being categorized into OA, RA, or other types of arthritis (including psoriatic arthritis, which is classified under other types).

### Definition of sarcopenia

In this study, we utilized the Examination Data from NHANES, the sum of lean mass in the extremities derived from DXA data was used to calculate the appendicular lean mass (ASM) for each participant. The skeletal muscle mass index (SMI) was calculated using the formula ASM/BMI. The diagnostic criteria for sarcopenia were based on the guidelines established by the Foundation for the National Institutes of Health (FNIH): an SMI value below 0.789 for males and below 0.512 for females. These criteria are consistent with the definitions used in other similar studies (22).

### Covariates

This study also evaluated demographic factors including age, gender, ethnicity, level of education (below high school, high school, college, and above college), and poverty-to-income ratio (PIR), with PIR categorized as < 1.3 for low income, 1.3–3.5 for middle income, and > 3.5 for high income. And based on previous factors related to NPAR, sarcopenia, and arthritis, other covariates were selected, including alcohol intake, smoking status, presence of chronic diseases, nutrient intake, and physical activity levels (10, 23). Alcohol intake was defined as drinkers (consuming more than 12 alcoholic beverages in a year) and non-drinkers (consuming fewer than 12 alcoholic beverages in a year). Diabetes was classified as diabetic, non-diabetic, and borderline based on physician diagnosis. Hypertension was determined as hypertensive or non-hypertensive based on personal responses and blood pressure measurements. Smoking status was classified as non-smokers (those who had never smoked 100 cigarettes in their lifetime), smokers (those who had smoked more than 100 cigarettes in their lifetime and had not quit), and ex-smokers (those who had smoked but had quit, with information on the timing of cessation available). Physical activity was categorized into exercisers and non-exercisers based on whether individuals engaged in vigorous or moderate physical activities. And other variables potentially relevant to the study content were included, such as total bone mineral density (TBMD), waist circumference, and several blood test indicators including lymphocyte percentage, monocyte percentage, hemoglobin, platelets, triglycerides, and total cholesterol, to observe whether there were any confounding relationships among them (4, 22).

### Statistical methods

Statistical analyses were performed utilizing R package (version 4.2.1) and EmpowerStats (version 2.0). For the data from 1999 to 2006, multiply imputed datasets provided by the NHANES database were utilized. For all years, data entries with missing values related to key indicator information, such as those required for the calculation of NPAR and SMI, were directly excluded. For other covariates with missing data, where the proportion of missing values was below 10%, different imputation methods were applied according to the variable type. Specifically, for continuous variables, mean imputation was used for those following a normal distribution, while median imputation was employed for those with a skewed distribution. For categorical variables, mode imputation was adopted. The weights were used and constructed according to the requirements on the NHANES website,^1^ so that the estimates can represent the US civilian non-institutionalized population across the combined survey cycles. The baseline characteristics of the study population were compared among the normal population, the arthritis population (subdivided into OA, RA, and other types of arthritis), and NPAR was categorized into quintiles (Q1–Q5) based on the sample size of the arthritis population (2,401 participants) to ensure balanced subgroup distribution, and to align with the dose-response relationship identified by RCS analysis. The cut-off values for each quintile were determined based on the actual distribution of NPAR in the study’s arthritis population. For continuous variables, weighted linear regression models were applied, while for categorical variables, weighted chi-square tests were used.

A multivariate logistic regression analysis was conducted to examine the association between NPAR and the prevalence of arthritis with sarcopenia, with the calculation of odds ratio (OR) and their corresponding 95% confidence interval (CI). Three models were utilized for the multivariate testing: Model 1 without any variable adjustment; Model 2 adjusted for gender, age, and race; and Model 3 adjusted for other covariates (excluding those related to the calculation of NPAR and SMI). Restricted cubic splines (RCS) were used to examine dose-effect relationships and assess non-linear associations. Subgroup analysis and interaction tests were conducted to observe the potential impact of other confounding factors. The diagnostic value of NPAR, as a composite factor, was compared with that of albumin or neutrophil percentage, individually, using the receiver operating characteristic (ROC) method for arthritis with sarcopenia. *P*-value < 0.05 was defined as statistically significant.

## Results

### Baseline characteristics of participant

The baseline characteristics of the subjects are presented in Table 1. We included 14,102 American adults in the study, among whom the prevalence of arthritis was approximately 17.03%, totaling 2,401 individuals. This included 782 with OA, 580 with RA, and 1,039 with other types of arthritis. We observed that the weighted mean age among individuals with arthritis (54.06 ± 13.50) was significantly higher than that of the non-arthritis population (39.63 ± 13.28), and the weighted proportion of females with arthritis (60.32%) was also notably higher than that of the non-arthritis population (48.03%) (*P* < 0.001). Among individuals with arthritis, the prevalence of sarcopenia was about 12.74%, with the highest prevalence in RA at 15.06%, which was significantly higher than that in the non-arthritis population (*P* < 0.001). The SMI values of individuals with arthritis (0.73 ± 0.19) were also generally lower than those of non-individuals with arthritis (0.83 ± 0.20) (*P* < 0.001). Additionally, the values of NPAR (13.81 ± 2.50) in the arthritis population were significantly higher than those in non-individuals with arthritis (13.24 ± 2.37) (*P* < 0.001).

**TABLE 1:** The baseline characteristics of participants.

Characteristic	No arthritis (*n* = 11,701)	Arthritis (*n* = 2,401)				*P*-value
		Total (*n* = 2,401)	OA (*n* = 782)	RA (*n* = 580)	Other (*n* = 1,039)	
Age (years)	39.63 ± 13.28	54.06 ± 13.50	54.75 ± 13.11	54.50 ± 14.04	53.27 ± 13.49	< 0.001
**Gender**						< 0.001
Male	6,029 (51.97%)	942 (39.68%)	284 (36.69%)	228 (41.21%)	430 (41.37%)	
Female	5,672 (48.03%)	1,459 (60.32%)	498 (63.31%)	352 (58.79%)	609 (58.63%)	
**Race**						< 0.001
Mexican American	2,231 (10.19%)	365 (4.87%)	75 (4.45%)	123 (7.16%)	167 (4.87%)	
Other Hispanic	1,134 (8.06%)	190 (5.87%)	41 (2.82%)	43 (5.47%)	106 (8.57%)	
Non-Hispanic White	4,146 (63.34%)	1,162 (73.42%)	455 (79.46%)	240 (67.08%)	467 (71.65%)	
Non-Hispanic Black	2,331 (10.79%)	492 (9.94%)	139 (7.87%)	135 (12.98%)	218 (10.11%)	
Other race	1,859 (7.62%)	192 (5.91%)	72 (6.4%)	39 (7.31%)	81 (4.80%)	
**Education**						< 0.001
Below high school	2,612 (16.31%)	727 (23.17%)	151 (14.77%)	229 (30.55%)	347 (26.34%)	
High school	2,545 (22.98%)	557 (25.95%)	162 (21.69%)	145 (30.80%)	250 (27.00%)	
University	3,539 (31.07%)	719 (30.42%)	286 (36.54%)	144 (24.62%)	289 (28.32%)	
Above university	3,005 (29.65%)	398 (20.46%)	183 (27.00%)	62 (14.03%)	153 (18.34%)	
**PIR**						0.009
< 1.3	3,269 (20.87%)	784 (23.42%)	226 (20.79%)	210 (27.14%)	348 (23.71%)	
1.3–3.5	3,916 (32.17%)	769 (32.28%)	258 (32.16%)	189 (34.20%)	322 (31.41%)	
> 3.5	4,516 (46.96%)	848 (44.3%)	298 (47.05%)	181 (38.65%)	369 (44.88%)	
**Physical examination**						
BMI	28.06 ± 6.29	30.23 ± 7.03	30.51 ± 7.35	29.20 ± 6.40	30.52 ± 7.02	< 0.001
Waist	95.57 ± 15.58	101.99 ± 16.22	102.47 ± 15.73	99.90 ± 16.05	102.63 ± 16.61	< 0.001
SMI	0.83 ± 0.20	0.73 ± 0.19	0.73 ± 0.19	0.73 ± 0.18	0.74 ± 0.19	< 0.001
Total BMD	1.12 ± 0.11	1.09 ± 0.13	1.09 ± 0.13	1.08 ± 0.13	1.10 ± 0.12	< 0.001
Total fat%	32.67 ± 8.49	36.82 ± 8.37	37.13 ± 8.29	36.27 ± 7.75	36.85 ± 8.72	< 0.001
**Sarcopenia**						< 0.001
No	10,631 (93.13%)	2,002 (87.26%)	671 (88.74%)	461 (84.94%)	870 (87.21%)
Yes	1,070 (6.87%)	399 (12.74%)	111 (11.26%)	119 (15.06%)	169 (12.79%)
**Smoke**						< 0.001
Current smoker	2,476 (22.32%)	633 (24.77%)	222 (24.71%)	151 (26.77%)	260 (23.81%)	
Non-smoker	7,126 (57.65%)	1,120 (45.10%)	344 (45.62%)	279 (43.70%)	497 (45.38%)	
Former smoker	2,099 (20.03%)	648 (30.13%)	216 (29.67%)	150 (29.53%)	282 (30.81%)	
**Alcohol intake**						< 0.001
Drinker	9,065 (80.67%)	1,771 (75.83%)	620 (80.12%)	395 (71.02%)	756 (74.72%)	
Non-drinker	2,636 (19.33%)	630 (24.17%)	162 (19.88%)	185 (28.98%)	283 (25.28%)	
**Diabetes**						< 0.001
Yes	730 (4.42%)	388 (12.28%)	113 (12.18%)	126 (15.18%)	149 (10.90%)	
No	10,805 (94.30%)	1,938 (85.11%)	637 (84.08%)	434 (82.25%)	867 (87.40%)	
Borderline	166 (1.28%)	75 (2.61%)	32 (3.74%)	20 (2.57%)	23 (1.70%)	
**Hypertension**						< 0.001
Yes	2,453 (19.32%)	1,132 (42.89%)	388 (46.76%)	279 (44.03%)	465 (39.15%)	
No	9,248 (80.68%)	1,269 (57.11%)	394 (53.24%)	301 (55.97%)	574 (60.85%)	
**Physical activity**						< 0.001
Yes	6,546 (61.93%)	1,260 (57.27%)	368 (51.89%)	329 (39.05%)	563 (59.83%)	
No	5,155 (38.07%)	1,141 (42.73%)	414 (48.11%)	251 (60.95%)	476 (40.17%)	
**Dietary intake**						
Total calories (kcal)	2,277.41 ± 1,035.10	2,063.84 ± 938.52	2,039.27 ± 876.86	2,049.91 ± 1,006.47	2,090.94 ± 951.17	< 0.001
Total protein (gm)	85.84 ± 43.92	78.46 ± 41.25	79.29 ± 41.46	78.83 ± 45.79	77.59 ± 38.57	< 0.001
Total carbohydrate (gm)	272.60 ± 134.47	247.16 ± 118.45	242.50 ± 113.56	248.19 ± 124.48	250.46 ± 119.13	< 0.001
Total fat (gm)	86.50 ± 48.10	80.32 ± 47.74	79.50 ± 44.88	77.81 ± 47.97	82.25 ± 49.78	< 0.001
**Blood test index**						
Lymph%	30.63 ± 7.92	29.72 ± 8.10	29.21 ± 7.98	30.14 ± 8.04	29.94 ± 8.20	< 0.001
Mono%	8.01 ± 2.14	7.93 ± 2.14	7.91 ± 2.11	7.92 ± 2.10	7.96 ± 2.18	0.119
Neut%	57.99 ± 8.89	58.96 ± 8.91	59.39 ± 8.81	58.61 ± 8.79	58.77 ± 9.03	< 0.001
HGB (g/dL)	14.40 ± 1.45	14.18 ± 1.36	14.17 ± 1.29	14.02 ± 1.45	14.25 ± 1.36	< 0.001
PLT (1,000 cells/uL)	250.20 ± 60.02	255.62 ± 66.14	251.07 ± 64.74	261.06 ± 74.74	256.63 ± 62.29	< 0.001
ALB (g/dL)	44.10 ± 3.37	43.12 ± 3.25	43.16 ± 3.30	43.00 ± 3.24	43.14 ± 3.22	< 0.001
CHOL (mg/dL)	192.66 ± 39.58	201.38 ± 39.48	202.31 ± 41.48	200.08 ± 36.90	201.26 ± 39.03	< 0.001
TG (mg/dL)	143.23 ± 124.77	160.79 ± 115.13	164.61 ± 131.48	159.71 ± 106.70	158.21 ± 104.26	< 0.001
NPAR	13.24 ± 2.37	13.81 ± 2.50	13.86 ± 2.54	13.90 ± 2.54	13.72 ± 2.45	< 0.001

The continuous variables were summarized using the mean ± standard deviation, whereas the categorical variables were characterized by unweighted counts of occurrences and weighted proportions. *P*-value was obtained by comparing the non-arthritis group with the overall arthritis population.

Among other variables, compared to non-individuals with arthritis, we discovered that individuals with arthritis tended to be non-Hispanic white, current or former smokers, have diabetes, hypertension, and engage in no physical activity. They also had lower educational levels (*P* < 0.001) and incomes (*P* = 0.009), as well as lower total energy intake (*P* < 0.001). Additionally, compared to non-individuals with arthritis, individuals with arthritis typically had higher, BMI, waist circumference, total fat content, neutrophil percentages, platelet counts, and triglyceride levels, as well as lower total BMD, albumin levels, lymphocyte percentages, and hemoglobin levels (*P* < 0.001).

The baseline characteristics of the studied arthritis participants, stratified by quintiles of NPAR, are presented in Table 2. Compared to participants in the lowest quintile (Q1) of NPAR values, those in the upper fifth quartile (Q5) had a higher prevalence of sarcopenia, a lower SMI, and were more likely to be female, have lower incomes, higher BMI, waist circumference, total fat content, hypertension prevalence, neutrophil percentage, and platelet count. Additionally, they had lower protein intake, lymphocyte percentage, monocyte percentage, albumin, hemoglobin, triglyceride levels, and engaged in less physical activity (*P* < 0.05).

**TABLE 2:** Baseline characteristics of participants stratified by quintiles of NPAR values.

Characteristic	Q1 (*n* = 477)	Q2 (*n* = 483)	Q3 (*n* = 478)	Q4 (*n* = 481)	Q5 (*n* = 482)	*P*-value
Age (years)	52.98 ± 13.03	53.88 ± 13.12	54.28 ± 14.41	54.58 ± 13.17	54.60 ± 13.55	0.3286
**Gender**						< 0.001
Male	206 (45.84%)	204 (42.95%)	179 (37.64%)	185 (38.75%)	168 (33.07%)	
Female	271 (54.16%)	279 (57.05%)	299 (62.36%)	296 (61.25%)	314 (66.93%)	
**Race**						< 0.001
Mexican American	62 (3.87%)	73 (4.00%)	83 (5.47%)	81 (5.55%)	66 (5.50%)	
Other Hispanic	40 (6.02%)	38 (6.32%)	34 (5.50%)	40 (6.40%)	38 (5.12%)	
Non-Hispanic White	181 (65.37%)	232 (73.99%)	261 (78.69%)	237 (73.25%)	251 (75.19%)	
Non-Hispanic Black	144 (15.88%)	98 (9.29%)	74 (6.99%)	85 (8.95%)	91 (8.91%)	
Other race	50 (8.87%)	42 (6.40%)	26 (3.35%)	38 (5.86%)	36 (5.28%)	
**Education**						0.389
Below high school	151 (24.34%)	145 (21.00%)	132 (22.92%)	156 (24.78%)	143 (23.11%)	
High school	95 (22.65%)	111 (26.79%)	114 (26.07%)	117 (25.61%)	120 (28.25%)	
University	134 (29.93%)	147 (29.49%)	149 (30.83%)	136 (29.77%)	153 (32.09%)	
Above university	97 (23.07%)	80 (22.72%)	83 (20.18%)	72 (19.84%)	66 (16.28%)	
**PIR**						0.006
< 1.3	134 (18.95%)	149 (19.91%)	155 (23.89%)	162 (25.38%)	184 (28.96%)	
1.3–3.5	152 (33.70%)	163 (31.56%)	144 (31.23%)	165 (33.39%)	145 (31.75%)	
> 3.5	191 (47.36%)	171 (48.52%)	179 (44.88%)	154 (40.77%)	153 (39.29%)	
**Physical examination**						
BMI	28.79 ± 6.01	29.41 ± 6.16	30.07 ± 6.66	31.01 ± 7.58	32.01 ± 8.19	< 0.001
Waist	99.14 ± 14.39	100.27 ± 15.64	101.48 ± 14.96	103.34 ± 15.99	106.08 ± 19.01	< 0.001
SMI	0.77 ± 0.21	0.76 ± 0.19	0.73 ± 0.19	0.72 ± 0.17	0.69 ± 0.17	< 0.001
Total BMD	1.09 ± 0.14	1.11 ± 0.12	1.09 ± 0.12	1.09 ± 0.12	1.08 ± 0.13	0.058
Total fat%	34.79 ± 8.50	35.95 ± 7.79	36.90 ± 8.35	37.72 ± 8.48	38.86 ± 8.19	< 0.001
**Sarcopenia**						< 0.001
No	430 (92.58%)	404 (88.56%)	406 (87.95%)	394 (87.74%)	368 (79.28%)	
Yes	47 (7.42%)	79 (11.44%)	72 (12.05%)	87 (12.26%)	114 (20.72%)	
**Smoke**						0.298
Current smoker	110 (23.74%)	121 (23.21%)	119 (22.72%)	126 (26.08%)	157 (28.53%)	
Non-smoker	233 (44.66%)	223 (44.83%)	225 (46.29%)	222 (43.49%)	217 (46.05%)	
Former smoker	134 (31.60%)	139 (31.96%)	134 (30.99%)	133 (30.44%)	108 (25.42%)	
**Alcohol intake**						0.987
Drinker	348 (75.83%)	358 (76.34%)	352 (75.59%)	356 (74.95%)	357 (76.35%)	
Non-drinker	129 (24.17%)	125 (23.66%)	126 (24.41%)	125 (25.05%)	125 (23.65%)	
**Diabetes**						0.059
Yes	63 (10.19%)	66 (10.95%)	76 (12.20%)	79 (11.49%)	104 (16.67%)	
No	397 (86.01%)	404 (86.61%)	385 (85.04%)	388 (86.20%)	364 (81.60%)	
Borderline	17 (3.80%)	13 (2.54%)	17 (2.54%)	14 (2.11%)	14 (1.73%)	
**Hypertension**						0.014
Yes	228 (45.12%)	208 (38.72%)	215 (40.58%)	234 (41.88%)	247 (48.78%)	
No	249 (54.88%)	275 (61.28%)	263 (59.42%)	247 (58.12%)	235 (51.22%)	
**Physical activity**						0.006
Yes	213 (39.91%)	226 (38.64%)	216 (42.18%)	230 (43.82%)	256 (49.65%)	
No	264 (60.09%)	257 (61.36%)	262 (57.82%)	251 (56.18%)	226 (50.35%)	
**Dietary intake**						
Total calories (kcal)	2,125.78 ± 900.87	2,020.79 ± 860.62	2,051.52 ± 1,002.95	2,124.77 ± 982.46	2,006.63 ± 933.70	0.162
Total protein (gm)	83.71 ± 44.07	75.55 ± 36.81	79.53 ± 42.16	77.98 ± 42.93	75.78 ± 39.81	0.018
Total carbohydrate (gm)	251.52 ± 119.04	244.36 ± 113.60	239.68 ± 119.09	257.45 ± 118.94	244.53 ± 121.04	0.177
Total fat (gm)	80.54 ± 43.10	78.36 ± 43.14	81.70 ± 53.06	84.59 ± 53.47	76.69 ± 44.43	0.121
**Blood test index**						
Lymph%	40.12 ± 6.52	32.84 ± 4.56	28.70 ± 4.16	25.48 ± 4.26	21.05 ± 4.92	< 0.001
Mono%	8.96 ± 2.31	8.24 ± 2.12	8.07 ± 1.89	7.51 ± 1.88	6.81 ± 1.81	< 0.001
Neut%	46.93 ± 6.03	55.18 ± 3.70	59.90 ± 3.65	63.80 ± 4.20	69.51 ± 5.58	< 0.001
HGB (g/dL)	14.35 ± 1.40	14.27 ± 1.27	14.28 ± 1.27	14.10 ± 1.34	13.86 ± 1.45	< 0.001
PLT (1,000 cells/uL)	251.19 ± 57.83	253.52 ± 60.63	247.88 ± 63.39	259.73 ± 67.88	267.06 ± 78.14	< 0.001
ALB (g/dL)	44.87 ± 3.05	44.14 ± 2.92	43.52 ± 2.55	42.54 ± 2.67	40.33 ± 3.07	< 0.001
CHOL (mg/dL)	208.30 ± 43.32	200.81 ± 35.11	202.99 ± 38.19	199.36 ± 38.87	195.18 ± 40.76	< 0.001
TG (mg/dL)	153.20 ± 105.32	155.85 ± 97.30	161.95 ± 142.51	166.73 ± 107.38	166.90 ± 115.09	0.239

Categorical variables shown as unweighted frequencies and weighted percentages.

### Associations between NPAR and sarcopenia among arthritis participants

The association between NPAR and the prevalence of sarcopenia among arthritis participants is presented in Table 3. When NPAR is analyzed as a continuous variable, it demonstrates a positive correlation with the prevalence of sarcopenia in arthritis, and this relationship remains significant even after adjusting for all potential confounding factors (OR: 1.22, 95% CI: 1.09, 1.36, *P* < 0.001). Specifically, for every one-unit increase in NPAR, associated with 22% higher odds of sarcopenia prevalence. When arthritis is stratified into OA, RA, and other types for analysis, and after adjusting for confounding factors, the positive correlation between NPAR and the prevalence of sarcopenia persists only in RA (OR: 1.36, 95% CI: 1.07, 1.74, *P* = 0.012) and other types of arthritis (OR: 1.37, 95% CI: 1.16, 1.63, *P* < 0.001), but not in OA (*P* = 0.884). When NPAR is treated as a categorical variable and stratified into quintiles (Q1–Q5), in the fully adjusted model, Q5 has a higher prevalence of sarcopenia compared to Q1 (Q5: OR: 3.47, 95% CI: 1.62, 7.44, *P* = 0.001). This finding is also applicable to RA (Q5: OR: 5.75, 95% CI: 1.11, 29.74, *P* = 0.037) and other types of arthritis (Q5: OR: 5.79, 95% CI: 1.79, 18.74, *P* = 0.003).

**TABLE 3:** Associations between NPAR and sarcopenia among arthritis participants.

Exposure	Total arthritis		OA		RA		Other arthritis	
	OR (95% CI)	*P*-value	OR (95% CI)	*P*-value	OR (95% CI)	*P*-value	OR (95% CI)	*P*-value
**Model 1**								
NPAR	1.13 (1.09, 1.18)	< 0.001	1.15 (1.06, 1.24)	< 0.001	1.08 (1.00, 1.17)	0.058	1.17 (1.09, 1.25)	< 0.001
**NPAR (quintile)**								
Q1	Reference	–	Reference	–	Reference	–	Reference	–
Q2	1.80 (1.22, 2.64)	0.003	3.71 (1.62, 8.50)	0.002	1.22 (0.63, 2.37)	0.551	1.60 (0.87, 2.94)	0.133
Q3	1.66 (1.12, 2.45)	0.012	2.34 (0.99, 5.52)	0.052	1.00 (0.49, 2.05)	1.000	2.01 (1.12, 3.63)	0.020
Q4	2.01 (1.38, 2.95)	< 0.001	3.78 (1.65, 8.69)	0.002	1.30 (0.68, 2.48)	0.426	2.01 (1.11, 3.64)	0.021
Q5	2.86 (1.98, 4.14)	< 0.001	4.37 (1.95, 9.79)	< 0.001	1.95 (1.05, 3.64)	0.035	3.17 (1.78, 5.64)	< 0.001
**Model 2**								
NPAR	1.14 (1.09, 1.20)	< 0.001	1.16 (1.06, 1.27)	0.001	1.05 (0.96, 1.15)	0.298	1.19 (1.10, 1.28)	< 0.001
**NPAR (quintile)**								
Q1	Reference	–	Reference	–	Reference	–	Reference	–
Q2	1.60 (1.06, 2.41)	0.024	3.51 (1.48, 8.28)	0.004	1.04 (0.49, 2.19)	0.921	1.39 (0.74, 2.63)	0.311
Q3	1.36 (0.90, 2.07)	0.144	1.94 (0.80, 4.73)	0.143	0.66 (0.29, 1.49)	0.316	1.84 (0.99, 3.42)	0.053
Q4	1.72 (1.15, 2.58)	0.009	3.07 (1.29, 7.30)	0.011	0.89 (0.42, 1.86)	0.754	1.98 (1.06, 3.68)	0.032
Q5	2.89 (1.95, 4.28)	< 0.001	4.29 (1.85, 9.94)	0.001	1.60 (0.79, 3.25)	0.193	3.54 (1.93, 6.50)	< 0.001
**Model 3**								
NPAR	1.22 (1.09, 1.36)	< 0.001	1.02 (0.81, 1.27)	0.884	1.36 (1.07, 1.74)	0.012	1.37 (1.16, 1.63)	< 0.001
**NPAR (quintile)**								
Q1	Reference	–	Reference	–	Reference	–	Reference	–
Q2	1.79 (1.08, 2.97)	0.024	2.87 (1.01, 8.15)	0.048	1.77 (0.65, 4.84)	0.265	1.51 (0.68, 3.34)	0.313
Q3	1.58 (0.89, 2.82)	0.119	0.98 (0.30, 3.28)	0.980	1.37 (0.40, 4.65)	0.615	2.59 (1.07, 6.29)	0.035
Q4	1.96 (1.03, 3.71)	0.039	1.37 (0.37, 5.06)	0.635	2.55 (0.70, 9.27)	0.156	3.02 (1.11, 8.26)	0.031
Q5	3.47 (1.62, 7.44)	0.001	1.83 (0.39, 8.54)	0.444	5.75 (1.11, 29.74)	0.037	5.79 (1.79, 18.74)	0.003

Model 1: adjusted for none; Model 2: adjusted for age, gender and race; Model 3: adjusted for gender, age, race, education, PIR, smoke, alcohol intake, diabetes, hypertension, physical activity, dietary intake, TBMD, waist, lymph%, Mono%, HGB, PLT, CHOL and TG. The bold values indicate *P* < 0.05.

**FIGURE 2:**
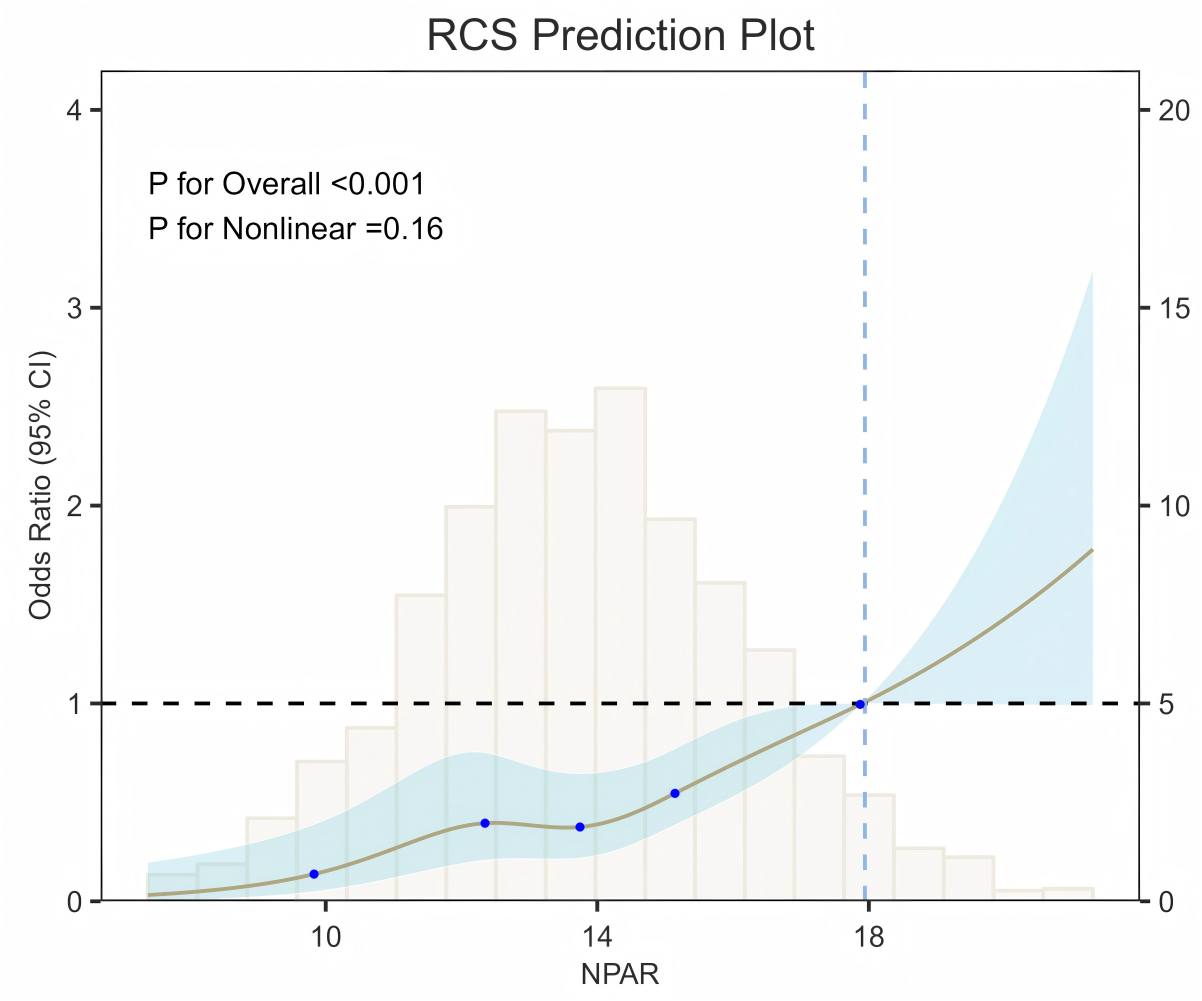
RCS for the association between NPAR and the prevalence of sarcopenia among arthritis participants (Brown line depicts the ORs, blue areas represent the 95% CI, and the cubic segments illustrate the distribution of the variable. The model was adjusted for gender, age, race, education, PIR, smoke, alcohol intake, diabetes, hypertension, physical activity, dietary intake, TBMD, waist, lymph%, Mono%, HGB, PLT, CHOL and TG).

In Figure 2, RCS indicated a dose-response relationship between the NPAR and sarcopenia. A positive relationship was observed among all participants (*P* for overall < 0.001, *P* for non-linear = 0.16).

### Subgroup analyses

**FIGURE 3:**
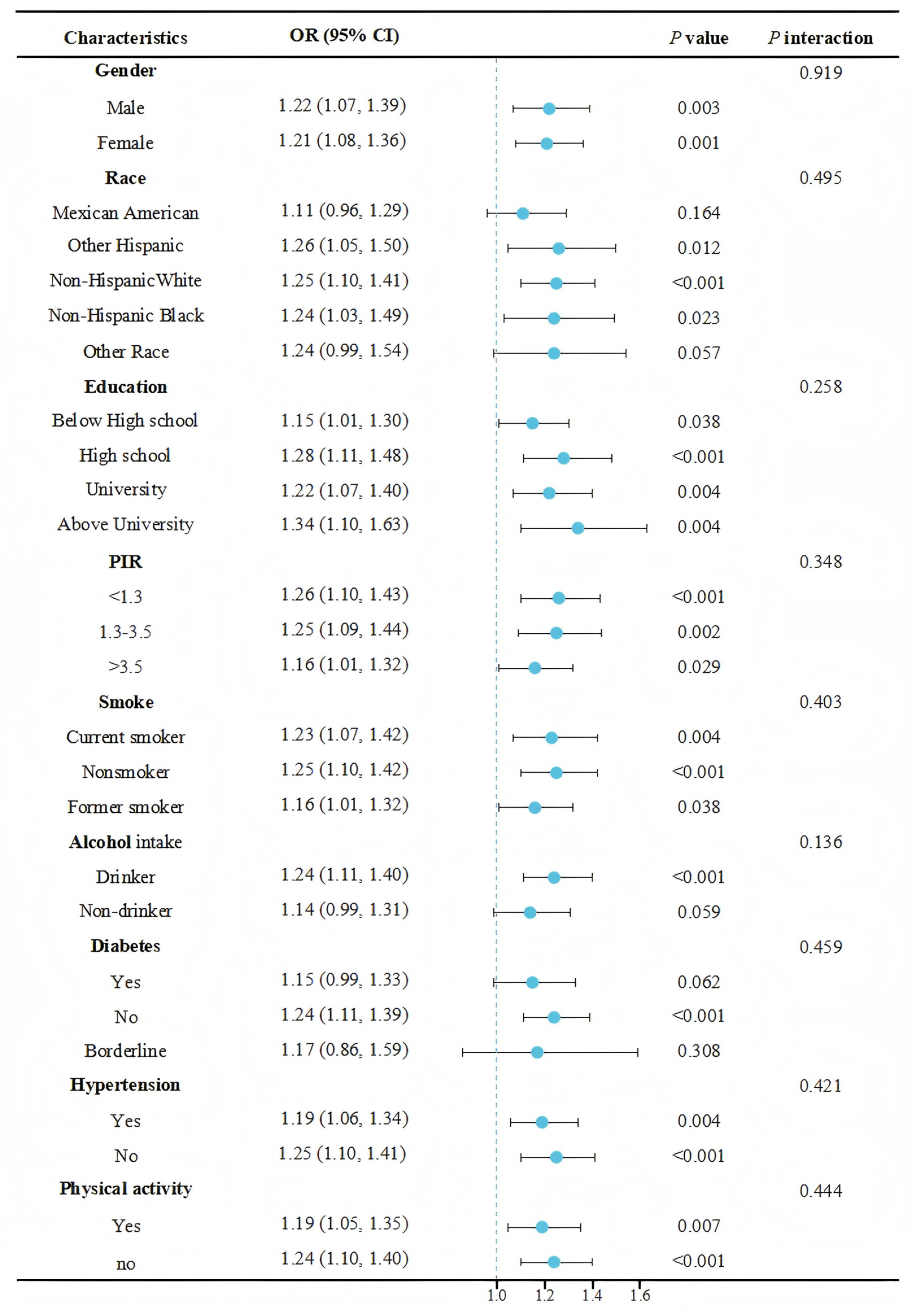
Forest plot of subgroup analysis for NPAR and prevalence of sarcopenia.

Subgroup analyses were performed on the basis of gender, race, education, PIR, smoke, alcohol intake, diabetes, hypertension and physical activity to assess the robustness of the association between NPAR with the prevalence of sarcopenia. In addition, we also analyzed the interactions among these variables. After adjusting for all possible potential confounders, the positive correlation between NPAR and the prevalence of sarcopenia among participants with arthritis persisted, except that no significant correlation was observed among Mexican Americans, individuals of other races, non-drinkers, diabetics, and those with borderline conditions (*P* > 0.05). Additionally, no significant interactions were observed among the other subgroups (*P* for interaction > 0.05) (Figure 3).

### The diagnostic predictive ability of NPAR for sarcopenia in arthritis participants

**FIGURE 4:**
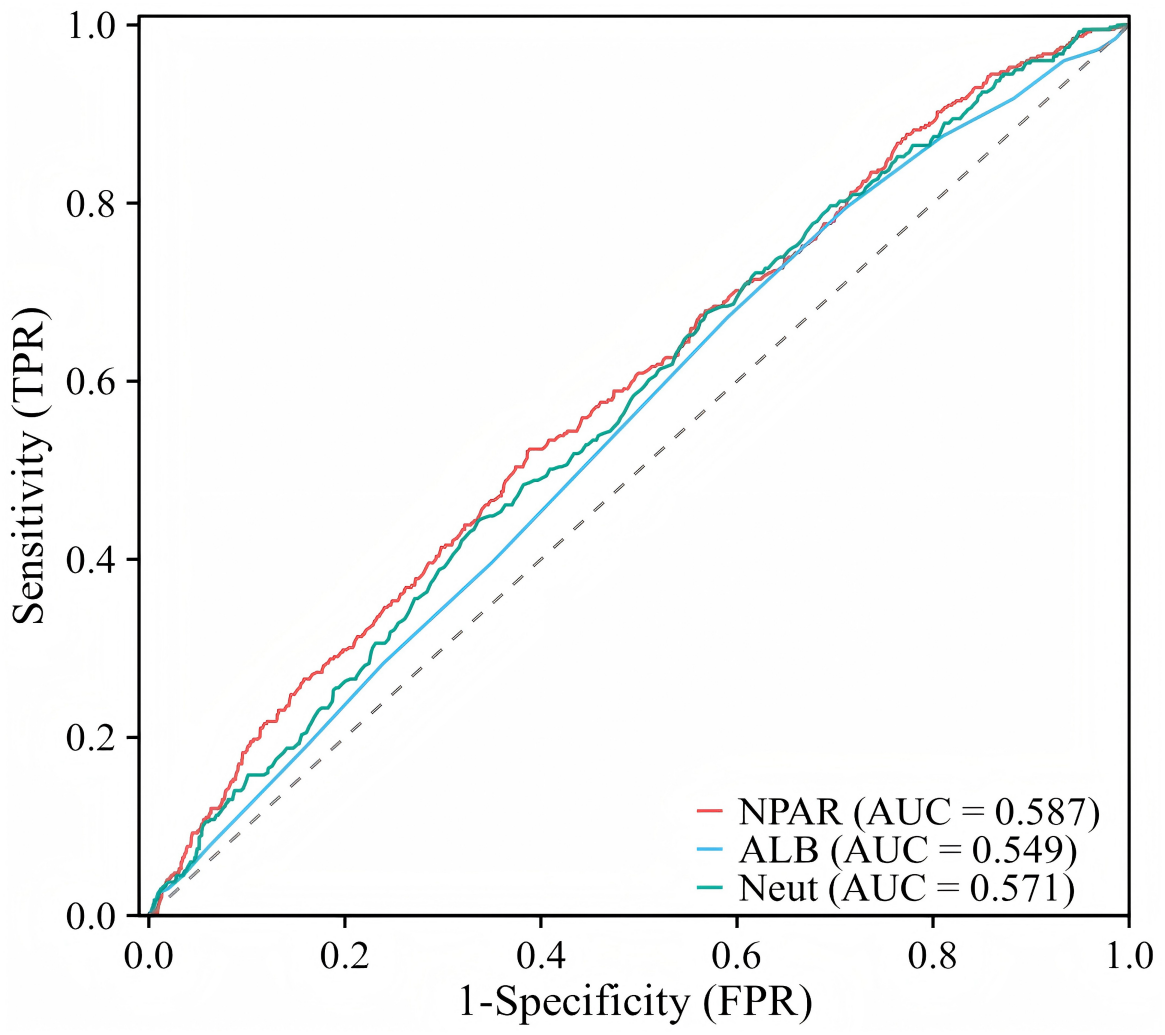
ROC curves and the AUC of NPAR, ALB and neutrophil percentage in diagnosing sarcopenia in arthritis participants.

In comparing the predictive abilities of NPAR, ALB and neutrophil percentage for sarcopenia in arthritis participants, we observed that NPAR demonstrated a certain advantage over ALB and neutrophil percentage. Specifically, as assessed by the AUC values derived from ROC curves, NPAR exhibited an AUC of 0.587 (95% CI: 0.557, 0.617), which was higher than that of ALB (AUC = 0.549, 95% CI: 0.519, 0.579) and neutrophil percentage (AUC = 0.571, 95% CI: 0.541, 0.601) (Figure 4). Given its AUC of 0.587, which remained below the 0.70 threshold generally required for standalone diagnostic utility, NPAR demonstrated only preliminary diagnostic predictive ability in this cohort of arthritis participants.

## Discussion

Based on the results of the aforementioned cross-sectional study, after adjusting for all potential confounding factors, NPAR remained significantly associated with the prevalence of sarcopenia among individuals with arthritis. However, this association was more pronounced in RA and other types of arthritis compared to OA, where it was less evident. To our knowledge, this is the first study to examine the relationship between NPAR and sarcopenia in individuals with arthritis.

Arthritis is often accompanied by sarcopenia, with patients with RA and OA both exhibiting a higher risk of developing sarcopenia (8, 24). The baseline of the study population supports this notion, showing that the overall prevalence of sarcopenia among individuals with arthritis is significantly higher than that in the non-arthritis population, with RA patients having the highest prevalence of sarcopenia. Additionally, individuals with arthritis had elevated levels of inflammatory markers such as neutrophil percentage and platelet count, while their SMI was significantly decreased. This may be related to the increased catabolism in individuals with arthritis due to their higher inflammatory levels. Chronic inflammation mediated by cytokines such as TNF and IL-6 can lead to abnormal muscle homeostasis, for instance, by exacerbating muscle protein catabolism (8). Baseline studies have also found that individuals with arthritis have higher BMI, total fat content, and waist circumference compared to healthy individuals. Obesity, particularly central obesity, affects the inflammatory processes in the body, as evidenced by increased circulating levels of pro-inflammatory proteins (25), leading to persistent inflammation and increased oxidative stress (23). Although moderate-intensity resistance training and high-intensity resistance training are important strategies for improving sarcopenia, many individuals with arthritis find it difficult to tolerate such treatment regimens due to their condition (26). This exacerbates the risk of sarcopenia in individuals with arthritis.

The detection of sarcopenia in individuals with arthritis holds significant importance. Currently, DXA is a crucial method for determining muscle mass, but its availability in primary care settings is limited (12). We did not choose to diagnose sarcopenia in individuals with arthritis based on grip strength, although grip strength is a key predictor of sarcopenia. This is because articular pain often limits muscle strength. The skeletal muscle atrophy associated with sarcopenia leads to decreased basal metabolic rate, reduced bone density, and increased risk of fractures in patients (27). Sarcopenia in individuals with arthritis often contributes to risks such as falls, which frequently result in adverse outcomes for these patients (28). Therefore, timely detection of sarcopenia is of great importance.

NPAR was first introduced in predicting mortality among patients with myocardial infarction (29),and the studies have found that patients in the high NPAR group exhibited higher in-hospital mortality rates and poorer outcomes. Subsequent applications in research on conditions such as cardiovascular diseases (30), depression (31), diabetes (32), chronic obstructive pulmonary disease (COPD) (33), psoriasis (34) and hepatobiliary diseases (35, 36), have demonstrated its significant predictive value for cardiovascular mortality and all-cause mortality (37).

Sarcopenia in individuals with arthritis is closely related to inflammation and nutritional status, and NPAR integrates both factors. Our study found that NPAR is significantly higher in individuals with arthritis compared to non-individuals with arthritis, which may be attributed to the higher levels of inflammatory response in individuals with arthritis. In multivariate regression analysis, NPAR was positively correlated with the prevalence of sarcopenia in the overall arthritis patient population. Within relevant subgroups, NPAR was associated with RA and other types of arthritis but not with OA. This may be related to the different pathogenesis of RA and OA. Although both conditions involve chronic inflammation, there are differences in pathogenesis and the degree of inflammation. In the context of RA, an autoimmune disorder, systemic inflammation is a common accompaniment. Beyond affecting small and large joints, RA also exhibits extra-articular manifestations (38). And the patients with RA often experience a decrease in skeletal muscle mass in their limbs when compared to individuals of normal weight, whereas their fat mass remains stable or may even increase (39). In contrast, OA, despite involving chronic inflammation, is predominantly viewed as a mechanically driven degenerative joint disease (40). The degree of inflammation in OA patients is generally lower than in those with RA, with most inflammatory markers in OA patients being normal or mildly elevated, in contrast to significant elevations of inflammatory markers like rheumatoid factor and anti-cyclic citrullinated peptide antibodies in RA patients (41). The number of infiltrating immune cells and the expression of cytokines in the synovial tissue of RA patients are higher than those in OA patients, and the inflammation levels in both are higher than in the normal population (42). A comparative study of synovial tissue samples from RA and OA patients revealed that RA patients exhibited increased lining hyperplasia, lymphocytic inflammation, neutrophils, and plasma cells. Conversely, OA patients were more commonly observed to have mast cells and the presence of fibrosis, while neutrophils were very rare (< 1%) in OA (43). The studies found that RA and OA exhibit distinct differences in various aspects, including cytokine profiles and immune cell phenotypes (41), differentially expressed genes in synovial membranes (44), and long non-coding RNAs in synovial fluid exosomes (45). From the angle of muscle catabolism induced by inflammation, sarcopenia associated with RA may exhibit a closer relationship with the extent of systemic inflammation when compared to OA. As an inflammatory marker, NPAR may better reflect the severity of inflammation in RA, thereby being associated with sarcopenia.

Although the exact mechanism linking NPAR and sarcopenia remains unclear, several studies have already identified potential associations between them. Previous research has found that low albumin levels may be an important risk factor for sarcopenia, and that albumin values are correlated with decreased muscle strength (46, 47). Additionally, persistent decreases in albumin may indicate ongoing inflammation in the body (48). Albumin also reflects the nutritional status of the body. A cross-sectional study found that, in addition to an increased risk of sarcopenia, RA patients often have poor nutritional status (49). NPAR combines both of these indicators, and ROC analysis has also shown that the AUC for the diagnostic accuracy of this composite indicator, NPAR, is higher than that of either indicator alone. This suggests that NPAR can be useful for screening for sarcopenia in individuals with arthritis and can also be used to observe changes in muscle mass index based on this indicator.

Our study has several strengths. Firstly, it is the first to evaluate the correlation between NPAR and the prevalence of sarcopenia in individuals with arthritis. Secondly, our analysis is based on a nationally representative sample, which provides a better representation of the health status of the overall population in the United States. Finally, our analysis takes into consideration potential confounding factors.

However, our study also has numerous limitations. Firstly, there may be many confounding factors affecting muscle mass and changes in NPAR levels in the body, and we were unable to identify and adjust for all potential factors. Secondly, the cross-sectional methodology inherently precludes the establishment of causal relationships between NPAR and sarcopenia in individuals with arthritis, and more related prospective studies are needed for validation. Thirdly, in the context of diagnostic prediction, the AUC analysis indicates that NPAR demonstrates insufficient diagnostic accuracy for standalone clinical determination of sarcopenia in individuals with arthritis. However, it may primarily serve as a complementary tool for preliminary screening and longitudinal monitoring of disease progression. Moreover, its integration into multivariate clinical diagnostic frameworks warrants consideration as a potential component variable. Additionally, in this study, a significant correlation between NPAR and sarcopenia was observed in RA and other types of arthritis, but the “other types” may include many and unknown subtypes, which may limit clinical application and necessitate further subclassification for targeted strategies. Furthermore, the sample size of the study population included was not sufficient, which may lead to bias in the results. Additionally, this study did not directly detect biomarkers of muscle catabolism. Future prospective studies could supplement the detection of such indicators to further verify the direct association between NPAR and the rate of muscle catabolism. Collectively, these limitations necessitate further clinical investigations to validate their operational efficacy.

## Conclusion

In summary, this study is the first to investigate the association between NPAR and the prevalence of sarcopenia in individuals with arthritis. Higher NPAR values may be associated with a higher odds of sarcopenia prevalence in individuals with arthritis, but these findings need further in-depth research for confirmation.

## Data Availability

The datasets presented in this study can be found in online repositories. The names of the repository/repositories and accession number(s) can be found in this article/supplementary material.

## Footnotes

1
https://wwwn.cdc.gov/nchs/nhanes/tutorials/weighting.aspx
